# Antiatherogenic and Cardioprotective Effects of a Xanthine Derivative KMUP-1 in ApoE Knockout Mice

**DOI:** 10.1155/cdr/8419343

**Published:** 2025-10-28

**Authors:** Erna Sulistyowati, Shang-En Huang, Chih-Chieh Hsu, Yi-Chia Wu, Yu-Ying Chao, Jong-Hau Hsu, Bin-Nan Wu, Zen-Kong Dai, Ming-Chung Lin, Jwu-Lai Yeh

**Affiliations:** ^1^Department of Medicine, Faculty of Medicine, Universitas Islam Malang, Malang, East Java, Indonesia; ^2^Department of Pharmacology, College of Medicine, Kaohsiung Medical University, Kaohsiung, Taiwan; ^3^Division of Plastic Surgery, Department of Surgery, Kaohsiung Medical University Hospital, Kaohsiung, Taiwan; ^4^Department of Surgery, School of Medicine, Kaohsiung Medical University, Kaohsiung, Taiwan; ^5^Regenerative Medicine and Cell Therapy Research Center, Kaohsiung Medical University, Kaohsiung, Taiwan; ^6^Department of Public Health, College of Health Sciences, Kaohsiung Medical University, Kaohsiung, Taiwan; ^7^Department of Pediatrics, Kaohsiung Medical University Hospital, Kaohsiung, Taiwan; ^8^Department of Pediatrics, College of Medicine, Kaohsiung Medical University, Kaohsiung, Taiwan; ^9^Department of Anesthesiology, Chi Mei Medical Center, Tainan, Taiwan; ^10^Department of Medical Laboratory Science and Biotechnology, Chung Hwa University of Medical Technology, Tainan, Taiwan; ^11^Department of Medical Research, Kaohsiung Medical University Hospital, Kaohsiung, Taiwan; ^12^Department of Marine Biotechnology and Resources, National Sun Yat-Sen University, Kaohsiung, Taiwan

**Keywords:** atherosclerosis, autophagy, cardiac remodeling, inflammation, KMUP-1

## Abstract

It is essential to manage cardiovascular disease related to atherosclerosis through understanding its disease progression mechanism. The effects of the xanthine derivative KMUP-1 on alleviating atherosclerosis and cardiac remodeling, as well as its underlying mechanisms, were examined. In this study, atherosclerosis and cardiac damage were induced in ApoE knockout (KO) mice by feeding them a high-fat diet (HFD) for 12 weeks. The co- and posttreatment of KMUP-1 was evaluated. Our results showed that KMUP-1 treatment significantly reduced body weight gain in HFD-induced mice. The Oil Red O and hematoxylin–eosin staining showed that KMUP-1 reduced the aortic plaque area, intima–media thickness, and intima–lumen thickness. KMUP-1 reduced inflammatory cytokines IL-1*β*, TNF-*α*, IL-6, and MCP-1 in the serum of mice through an ELISA assay. Moreover, echocardiography evaluation indicated that KMUP-1 attenuated left ventricular cardiac hypertrophy and restored cardiac function. Further, KMUP-1 treatment suppressed proapoptotic protein Bax and reversed Bcl-2 level by promoting autophagy-related Gene 7 and autophagosome marker LC3-II activation in the vascular through immunofluorescence and western blotting assay. KMUP-1 improved the serum lipidomic profile. Both co- and posttreatment of KMUP-1 stimulated autophagy and reduced inflammation and apoptosis, against atherosclerosis and cardiac remodeling in an ApoE-KO mouse model. It suggests its potential as a therapeutic agent for cardiovascular diseases.

## 1. Introduction

Atherosclerosis is the chronic buildup of plaque on arterial walls, driven by inflammation, lipid accumulation, calcification, vascular smooth muscle cell (VSMC) proliferation, and matrix remodeling, leading to the narrowing of the arteries [1]. Disrupted lipid metabolism and inflammation play key roles in atherosclerosis. Several risk factors like aging, diabetes, hypertension, high cholesterol, and smoking accelerate its progression. Certain biomarkers also signal inflammation-linked cardiovascular risk, even in those without classic risk factors [2]. Studies show that human biomarkers can predict cardiovascular risk from inflammation, even in people without evident disease or traditional risk factors. Impaired autophagy, the loss of the ability to degrade cellular components, may contribute to inflammation and atherosclerosis progression, highlighting the importance of maintaining cellular balance through autophagy [3].

Atherosclerotic plaque formation involves lipid-triggered activation of inflammatory cytokines (IL-1*β* and TNF-*α*), leading to deposition in the vascular walls and subsequently promoting the recruitment of immune cells that eventually form foam cells by binding to lipoproteins [1, 2]. Additionally, impaired autophagy has been linked to atheroma formation by affecting various cell types and contributing to atherosclerotic plaque development through dysregulated autophagy in vascular tissues [4, 5]. Recent studies indicate that impaired autophagy promotes atherothrombosis by inducing senescence and apoptosis in endothelial cells [6], necrosis and apoptosis in macrophages [7], and senescence in VSMCs [8]. Preliminary works focused on autophagy markers like p62 and LC3-II [9], which are detected in plaque-derived cells from both animals and humans. Notably, LC3-II levels are lower in patients with unstable plaques compared to those with stable ones [10]. Deficiency of autophagy proteins like Autophagy-Related 7 (Atg7) [11, 12] or Atg5 [13] in VSMCs promotes atheroma development. Atg7, a key autophagy gene regulating p53 activity, may link autophagy to cell cycle control [14]. Additionally, microtubule-associated light chain protein LC3 is key to autophagy initiation; the LC3-I converts to LC3-II by conjugation with phosphatidylethanolamine, facilitating autophagosome formation [15]. Autophagy deficiency in VSMCs accelerates senescence, and it contributes to diet-induced atherogenesis, while indicating the essential role of basic autophagy in cell survival and plaque stability [16]. Furthermore, in atheromasia, TNF-*α* secreted by inflammatory VSMCs stimulates both apoptosis and autophagy [17, 18]. When cholesterol crystals persist in plaques, they trigger IL-1*β* secretion, resulting in macrophage inflammasome hyperactivation [19]. These findings collectively highlight the role of autophagy in vascular health and its implications in atherosclerosis progression.

Our previous study showed that self-developed xanthine-based KMUP-1 (7-[2-[4-(2-chlorophenyl) piperazinyl]ethyl]-1,3-dimethylxanthine) protects against liver damage and apoptosis in a rat model of hepatic ischemia–reperfusion injury by reducing oxidative stress and inflammation. KMUP-1, as a nitric oxide donor, increases cyclic GMP/Protein Kinase G signaling and decreases vascular endothelial growth factor in hypoxic hepatocytes, contributing to its hepatoprotective effects [20]. A recent study revealed that KMUP-1 exerts antilipidemic effects by reducing plasma low-density lipoprotein (LDL), increasing high-density lipoprotein (HDL), and promoting the hydrolysis of triglycerides (TGs) for hepatic fat reduction. This effect is achieved through the activation of Protein Kinase A and G, along with hormone-sensitive lipase-mediated lipolysis in high-fat diet (HFD) mice, suggesting its potential for treating obesity and inflammatory fatty liver [21]. Based on the above research, we hypothesized that impaired autophagy, increased inflammation, and cardiac remodeling occur in our hyperlipidemia and atherosclerosis mouse model, whereas KMUP-1 treatment improves autophagy, suppresses inflammation, and restores cardiac function. We validated these hypotheses and identified a new insight by which KMUP-1 confers cardiovascular protection.

## 2. Methods and Materials

### 2.1. Animal Care and Preparations

To create an animal model for atherosclerosis, we utilized Apolipoprotein E–knockout (ApoE-KO) mice, as previously studied, as they bear a close resemblance to the human disease [22]. Eight weeks of 32 male ApoE-KO mice with C57BL/6 J genetic backgrounds (22 ± 2 g) were provided by the Jackson Laboratory (Bar Harbor, ME, United States). Mice were housed in the animal facility at Kaohsiung Medical University, with a 12-h day/night cycle. The ARRIVE guidelines 2.0 (published by the National Institutes of Health) were pursued for the animal procedures. The study was approved by the Institutional Animal Care and Use Committee (IACUC) of Kaohsiung Medical University with Approval Number IACUC 101183. The research protocol was designed to cause no harm to the animal and was made smaller for the number of mice to achieve statistical significance and reproducibility of the experiments.

During 1 week of acclimatization in the animal house, food and water were given ad libitum to the mice. Further, the light cycle, temperature, and humidity were also under control. Before the experiment, a standard diet (STD) (MF; Oriental Yeast, Tokyo, Japan) was given to the mice for one night and then switched to an HFD and then split into two control (CTL) and two treatment groups at random to create four groups. The HFD used a western diet containing 0.15% cholesterol and 21% fat (wt/wt) (F2WTD; Oriental Yeast). Eight mice were used in each group. The CTL or positive CTL mice received either STD or HFD, and the treatment group was fed an HFD with KMUP-1 (5 mg/kg/day) dissolved in double-distilled water administered by oral gavage. In the 12-week experiment, the cotreatment group was fed an HFD and 5 mg/kg/day KMUP-1 for the full 12 weeks. The posttreatment group was fed an HFD from Weeks 1 to 8 and then switched to STD until Week 12. KMUP-1 (5 mg/kg/day) was given from Weeks 9 to 12. We performed the experiments on all mice to ensure robust scientific data while considering practical costs and animal welfare. Based on our previous studies in several rodent models, a KMUP-1 dose of 5 mg/kg/day is appropriate and well tolerated, showing adverse effects on animals [23–25]. Every week, we recorded the body weight of the mice. After Week 12, all animals were sacrificed, followed by structural and biochemical analysis.

### 2.2. Echocardiography

For the evaluation of cardiac morphology, we used a Vevo 2100 high-resolution in vivo imaging system (Fujifilm, Tokyo, Japan) by the end of the study. As a previous method, we anesthetized the mice with isoflurane (Panion & BF Biotech Inc., Taipei, Taiwan), and then the echocardiogram images were taken. We ensured random assignment of 32 male ApoE-KO mice, and sonographers were blinded to the treatment groups to prevent observer bias. Five consecutive cardiac cycles were used to calculate each measurement. The anesthetized mice were positioned on a board. Consequently, the skin on the thorax of the mice was shaved to facilitate sonography. Then EcoGel 100 (Eco-Med Pharmaceutical Inc., Mississauga, Ontario, Canada), an ultrasound gel, was applied to it. We used a 40 MHz echocardiography probe to get an M-mode trace of the left ventricle, facilitating the left ventricular internal dimension (LVID) measurement at both end-diastolic and end-systolic phases (LVIDd and LVIDs), as well as the left ventricular posterior wall at end-systole (LVPWs) [26].

### 2.3. Blood and Serum Sample Preparation

After the experiment, approximately 1 mL of blood was obtained through heart puncture and transferred to serum biochemical tubes. Subsequently, to separate serum for biochemical analysis, the blood samples were centrifuged at 3000 rpm at 4°C for 10 min. Supernatants were gathered and separated into tubes, then kept at −80°C. The serum concentrations of mouse IL-1*β*, TNF-*α*, IL-6, and MCP-1 were assessed utilizing ELISA assay kits (Catalog # DY401, DY410, DY406, and DY479; R&D Systems, Minneapolis, MN, United States) in accordance with the manufacturer's guidelines [27]. The lipid profile was assessed by measuring levels of total cholesterol (TC), TG, LDL, and HDL. The Roche cobas c 311 biochemical analyzer (Roche Diagnostics, Switzerland) was used to carry out the determinations of TC, TG, LDL, and HDL. The aorta was swiftly extracted in a chilled environment and prepared for histopathological examination or biochemical assessment.

### 2.4. Histopathology Analyses

The segments of the thoracic aorta and hearts were preserved in a 10% paraformaldehyde solution (Sigma-Aldrich, Saint Louis, MO, United States), after which they were embedded in paraffin. The vascular tissue and hearts that were embedded were sliced into sections of 5 *μ*m. Following dewaxing with xylene and hydration through a sequence of graded ethanol concentrations, the hematoxylin (Sigma-Aldrich, Saint Louis, MO, United States) staining was carried out for 2 min as per the earlier method [26, 28]. Following a rinse with tap water, the sections mentioned above were then stained with eosin (Sigma-Aldrich) for 3 min. Later, the sections were washed, dehydrated, and soaked in xylene. Afterward, the sections underwent microscopic observation.

The thickness of the arterial media was assessed by measuring the distance from the internal elastic lamina to the external elastic lamina. Measurements from four distinct points—two of which are crossed to make a perpendicular angle—were averaged for each slide. Two distinct sites were used to define the lumen's inner diameter (two linked lines created a perpendicular angle). The media-to-lumen ratio was determined using the recorded inner diameter of the lumen and media data. The surfaces of Oil Red O–positive lesions on the en face preparation of the entire aorta were quantified to measure atherosclerotic lesions. The sections were obtained with a Nikon Eclipse TE 2000-S (Tokyo, Japan) fitted with a Nikon DS-Fi1 digital camera and NIS-Element F Version 3.0 software for image evaluation.

### 2.5. Immunofluorescence Staining

To perform immunofluorescence staining, we initially froze the aortic root in an optimum cutting temperature compound to define the expression and distribution of Atg7 protein activation in the aorta and then cut it into 4-*μ*m-thick sections. The primary antibodies, rabbit polyclonal anti-*α*-smooth muscle actin (Catalog Number: 19245, Cell Signaling; 20 *μ*g/mL) and rabbit polyclonal anti-Atg7 (Catalog Number: 8558, Cell Signaling; 20 *μ*g/mL), were used and then incubated with the secondary antibodies, FITC- or TRITC-conjugated anti-rabbit IgG (Santa Cruz Biotechnology, United States), respectively. The cells' nucleus was colored using 4⁣′,6-diamidino-2-phenylindole (DAPI, Beyotime, Beijing, China). Fluorescent images were captured using a confocal laser-scanning microscope (Zeiss LSM 700, Carl Zeiss MicroImaging GmbH, Jena, Germany), and the number of nuclei labeled by smooth muscle *α*-actin (SM*α*) was automatically counted in each field with an image analysis software (Zeiss AxioVision software) [29]. The average fluorescence intensity of Atg7 was calculated by dividing the fluorescence value of all pixels in six randomly chosen aortic sections by the total pixel count, as obtained through confocal microscopy with consistent laser and photomultiplier configurations.

### 2.6. Western Blotting

The aorta tissue was homogenized and subsequently centrifuged at 13,000 rpm for 30 min at 4°C to collect the supernatant. The preparation of aorta tissue extracts was performed using a lysis buffer that contained 20 mM Tris-HCl, 1 mM dithiothreitol (DTT), 5 mM EGTA, 0.5 mM PMSF, 20 *μ*M leupeptin, and 20 *μ*M aprotinin. The total protein was separated by sodium dodecyl sulfate–polyacrylamide gel electrophoresis (SDS-PAGE) on 10%–12% acrylamide gels, followed by transfer to a polyvinylidene difluoride (PVDF) membrane. The PVDF membranes underwent blocking for 1 h using 5% fat-free milk. We then incubated the membranes with the respective primary antibodies overnight at 4°C [27]. We used the primary antibodies as follows: anti-Bax (Catalog Number: GTX109683, GeneTex; 1 *μ*g/mL), anti-Bcl-2 (Catalog Number: SAB4500003, Sigma-Aldrich; 1 *μ*g/mL), anti-Atg7 (Catalog Number: 8558, Cell Signaling; 2 *μ*g/mL), anti-LC3 (Catalog Number: 2775, Cell Signaling; 2 *μ*g/mL), and *β*-actin as the internal loading control (Catalog Number: A5441, Sigma-Aldrich; 0.5 *μ*g/mL). We observed the blots with an enhanced chemiluminescence (ECL) system from Merck Millipore (Billerica, United States), and the gray values were measured using ImageJ software.

### 2.7. Statistical Analyses

The data were shown as mean ± standard error of mean (SEM). For mouse body weight, the effects of diet, KMUP-1 cotreatment, and KMUP-1 posttreatment were tested using two-way analysis of variance (ANOVA). Normal distribution and homogeneity of variance were assessed using the Shapiro–Wilk test and Bartlett's test, respectively. A significant interaction between diet, co-, and posttreatment was observed, followed by post hoc Fisher's least significant difference (LSD) test. Other data satisfying both normality and equal variance criteria were analyzed using one-way ANOVA, followed by post hoc Fisher's LSD test for multiple comparisons. Differences with a *p* value of 0.05 or less were considered statistically significant. Additionally, we conducted data analysis using SPSS software Version 26.0 (IBM, Armonk, United States). The graphs were created with SigmaPlot software Version 12.0 (Grafiti LLC, United States).

## 3. Results

### 3.1. KMUP-1 Reduces Atherosclerotic Lesion Formation in ApoE-KO Mice

**Figure 1:**
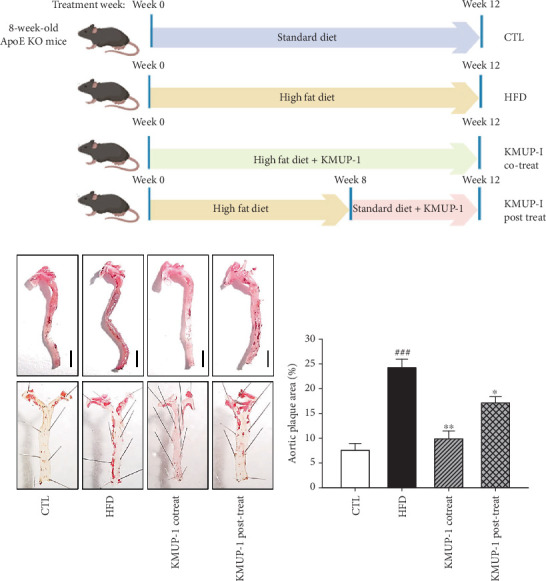
KMUP-1 reduces the formation of atherosclerotic lesions in ApoE-KO mice. (a) Outline of the animal experimental protocol. ApoE-KO mice were randomly divided into four groups: control (CTL) group, high-fat diet (HFD) group, cotreatment KMUP-1 (KMUP-1 cotreat) with HFD group, and posttreatment KMUP-1 (KMUP-1 posttreat) with HFD group. (b) Representative photographs of the aorta and the en face method performed using Oil Red O staining. (c) Quantification of the aortic plaque area. All scale bars indicate 5 mm. Values were represented as mean ± SEM, *n* = 8. ⁣^###^*p* < 0.001 versus CTL group. ⁣^∗^*p* < 0.05 and ⁣^∗∗^*p* < 0.01 versus HFD group.

Apolipoprotein E (ApoE) is essential for removing cholesterol-rich lipoproteins, and ApoE knockout (KO) animals exhibit disrupted cholesterol metabolism and accelerated neointima formation [30]. We examined whether KMUP-1 inhibits atherosclerotic formation in ApoE-KO mice. To define whether our experiment in treating a HFD was done, we compared the CTL group, which provided a STD, with the HFD group.  Figure 1a shows the experimental protocol outline in this study. HFD feeding mice significantly increased the percentage of the aortic plaque area compared to the CTL group (Figure 1b,c; *p* < 0.001). Furthermore, to determine whether KMUP-1 treatment benefits cardiovascular diseases in rats fed an HFD, or after the HFD was stopped, we established two different KMUP-1 treatment groups: cotreat and posttreat. Histologically, the whole aorta showed Oil Red O–positive lesion regions that were significantly reduced in both the cotreat (*p* < 0.01) and posttreat (*p* < 0.05) groups, in comparison to the HFD group (Figure 1b). Moreover, quantifying the aortic plaque area is a reliable indicator of atherosclerotic lesion progression. Consistently, the percentage of the aortic plaque area was reduced in the cotreat and posttreat groups (Figure 1c). These results suggest that administering KMUP-1 at 5 mg/kg/day may ameliorate atherosclerotic lesions in ApoE-KO mice fed an HFD.

Furthermore, we found that body weight was no noticeable difference among the four groups at baseline. At Week 12, before sacrifice, body weight gain was higher in HFD-fed ApoE-KO mice compared to the CTL group (*p* < 0.001; Supporting Information 1: Figure S1A,B), and after KMUP-1 treatment (both co- and posttreat), the body weight of the mice was decreased compared to the HFD group (*p* < 0.01; Supporting Information 1: Figure S1A,B). Referring to previous research [21], our findings reveal that an HFD elevates body weight gain, while KMUP-1 treatment helps to restrain it.

### 3.2. KMUP-1 Reduces Intimal Hyperplasia in the Aortic Sinus and Has Anti-Inflammatory Effects

**Figure 2:**
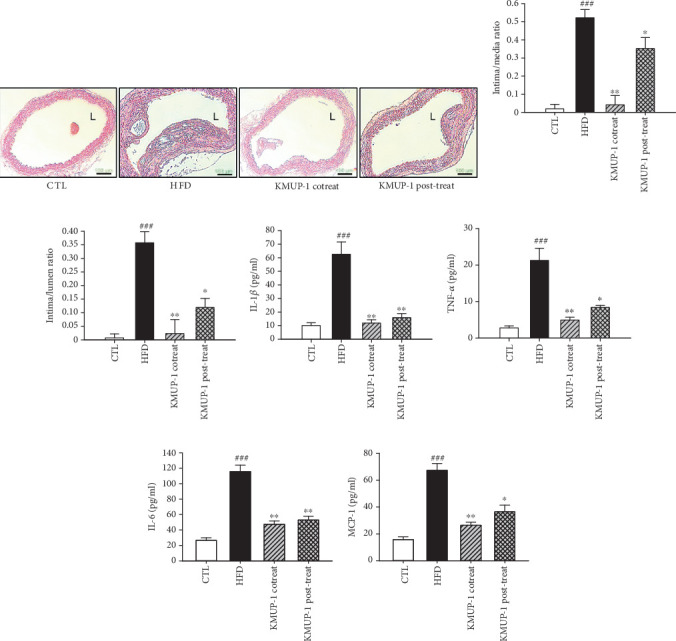
KMUP-1 reduces intimal hyperplasia in the aortic sinus and has anti-inflammatory effects. (a) Representative photomicrographs of arterial cross-sections were performed using hematoxylin and eosin staining. (b, c) Quantification of (b) aortic intima/media and (c) intima/lumen ratios. (d–g) Quantification of proinflammatory cytokines of (d) IL-1*β*, (e) TNF-*α*, (f) IL-6, and (g) MCP-1 in the mice serum. L means lumen. All scale bars indicate 100 *μ*m. Values were represented as mean ± SEM, *n* = 8. ⁣^###^*p* < 0.001 versus CTL group. ⁣^∗^*p* < 0.05 and ⁣^∗∗^*p* < 0.01 versus HFD group.

We assessed the effect of KMUP-1 on intimal hyperplasia. Hematoxylin and eosin staining of the aortic sinus showed extensive hyperplasia on the intimal layer of the HFD group versus the CTL group (Figure 2a). Cotreatment and posttreatment of KMUP-1 lowered the intimal layer in ApoE-KO mice fed with HFD. To define whether atherosclerosis was detected in the aorta, we measured the ratio of intima to media thickness and the ratio of intima thickness to lumen diameter. As shown in Figure 2b,c, HFD significantly increases the intima to media ratio and the intima to lumen ratio (*p* < 0.001). Treatment with KMUP-1, both as a co- and posttreatment, affected the reduction of the intima to media ratio and intima to lumen ratio (*p* < 0.05). The proatherogenic roles of proinflammatory cytokines are well established and have a profound influence on the pathogenesis [31]. We subsequently identified the expression of proinflammatory cytokines IL-1*β*, TNF-*α*, IL-6, and MCP-1 in the serum of the mice. Figures 2d, 2e, 2f, and 2g show results that HFD feeding significantly increased these proinflammatory cytokines (*p* < 0.001). Further, KMUP-1 co- and posttreatment reduced the serum levels of IL-1*β*, TNF-*α*, IL-6, and MCP-1 of ApoE-KO mice fed an HFD, indicating its anti-inflammatory properties.

HFD exposure not only induces atherosclerosis but also alters cardiac parameters in rodent models [32]. Therefore, our current study additionally demonstrated that KMUP-1 treatment may diminish cardiac hypertrophy in ApoE-KO mice fed with HFD (Supporting Information 2: Figure S2A). The ratio of whole heart to body weight and whole heart to tibial length showed that HFD possessed a higher ratio than the CTL (*p* < 0.001; Supporting Information 2: Figure S2B,C). Treatment with KMUP-1 (co- and posttreatment) significantly decreased both ratios in ApoE-KO mice fed an HFD. Following the echocardiographic profile of mice, KMUP-1 treatment affects cardiac function in mice fed with HFD. M-mode echocardiogram images of the mice before sacrifice are shown in Supporting Information 3: Figure S3A. The HFD showed a notable rise in the left ventricular end-diastolic dimension (LVEDD) (*p* < 0.05) and the left ventricular end-systolic dimension (LVESD) (*p* < 0.05) when compared to the CTL group. KMUP-1 co- and posttreatment suppressed HFD-enhanced LVEDD and LVESD (*p* < 0.05; Supporting Information 3: Figure S3B,C). The lowest values of left ventricular parameters, including fractional shortening (FS) (*p* < 0.01) and ejection fraction (EF) (*p* < 0.05), were found in HFD mice compared with the CTL group. KMUP-1 co- and posttreatment restored FS and EF values (*p* < 0.05; Supporting Information 3: Figure S3D,E). As shown in Table 1, our results indicate that KMUP-1 improved left ventricular function in ApoE-KO mice fed with HFD. These results are in association with our previous studies [33, 34], which showed that xanthine derivatives exhibit myocardial cell protection and repress aortic aneurysm formation.

### 3.3. The Proautophagy Effect of KMUP-1 Targeting Atg7 Activation in Atherosclerosis Mice

**Figure 3:**
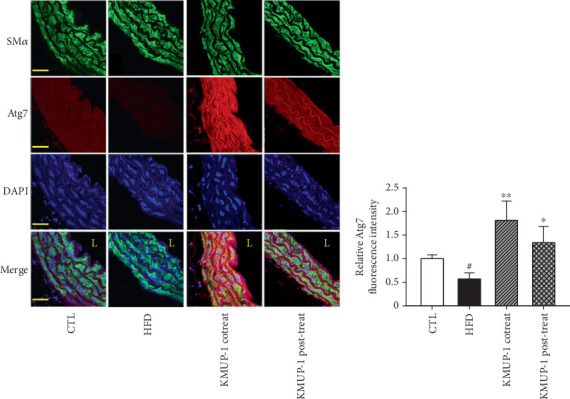
The antiatherosclerotic effects of KMUP-1 targeting Autophagy-Related 7 protein activation in the HFD-induced ApoE-KO mice. (a) Representative immunofluorescence images of arterial sections were stained for smooth muscle *α*-actin (green) and Atg7 (red). (b) Quantification of relative Atg7 fluorescence intensity. L means lumen. The nuclei were stained with DAPI. All scale bars indicate 100 *μ*m. Values were represented as mean ± SEM, *n* = 5. ⁣^#^*p* < 0.05 versus CTL group. ⁣^∗^*p* < 0.05 and ⁣^∗∗^*p* < 0.01 versus HFD group.

We conducted immunofluorescence staining of arterial sections to examine the impact of KMUP-1 on autophagy regulation in ApoE-KO mice fed a HFD. We initially stained for SM*α* on cross-sections of mice with aortic dissections (Figure 3a, green). We noticed a clear accumulation of SM*α*^+^ cells from the intima to the adventitia layer of the aortic wall in HFD mice. Next, the aortic sections were stained with the antibody against Atg7 (red).  Figure 3a,b indicates that the relative Atg7 fluorescence intensity was reduced in the HFD group (*p* < 0.05) compared to the CTL group. The cotreatment (*p* < 0.01) and posttreatment (*p* < 0.05) of KMUP-1 can both cause an increase in the relative Atg7 fluorescence intensity of aorta sections. This means KMUP-1 possesses a proautophagy effect by targeting Atg7 protein activation against atherosclerosis in HFD-fed ApoE-KO mice.

### 3.4. The Proautophagy Effect of KMUP-1 Offers Antiapoptosis Properties in Atherosclerosis Mice

**Figure 4:**
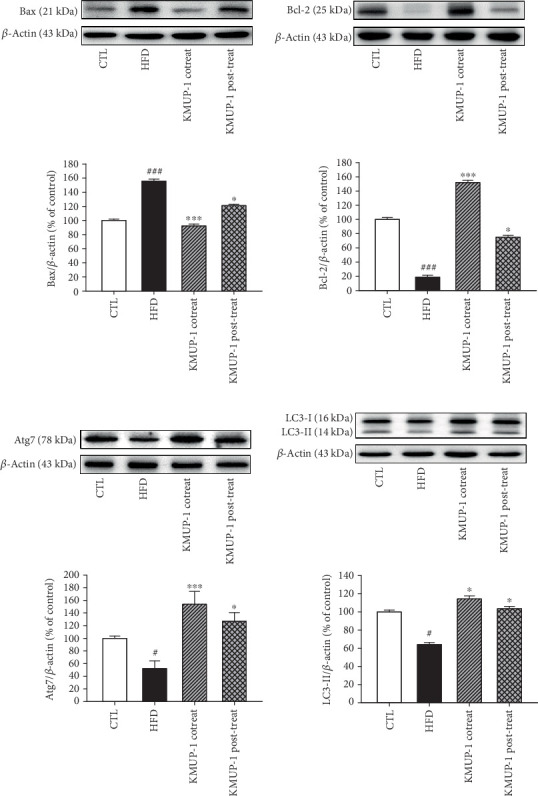
The antiapoptotic effects of KMUP-1 are promoted by autophagy in the HFD-induced ApoE-KO mice. (a, b) The apoptotic protein expressions of (a) Bax and (b) Bcl-2 were examined by western blot and quantitatively analyzed. (c, d) The autophagic protein expressions of (c) Atg7 and (d) LC3 were examined by western blot and quantitatively analyzed. Values were represented as mean ± SEM, *n* = 5. ⁣^#^*p* < 0.05 and ⁣^###^*p* < 0.001 versus CTL group. ⁣^∗^*p* < 0.05 and ⁣^∗∗∗^*p* < 0.001 versus HFD group.

A complex interplay exists between apoptosis and autophagy, highlighting their inverse relationship in atherosclerosis-related cardiovascular disease [35]. To determine whether the proautophagy effect of KMUP-1 plays a protective role against apoptosis, we evaluated the expression of Bax and Bcl-2, the apoptosis-associated proteins in the aortic tissue of ApoE-KO mice. Our findings showed that HFD-fed markedly increased the percentage of expression of Bax, a proapoptotic protein, from CTL to 155.90 ± 2.90 and 100.00% ± 2.10%, respectively (*p* < 0.001, Figure 4a). Both cotreatment and posttreatment of KMUP-1 decreased the percentage of expression of Bax, 92.30% ± 2.50% (*p* < 0.001) and 121.00% ± 1.90% (*p* < 0.05), respectively, compared to HFD mice.  Figure 4b shows that HFD-fed significantly decreased the percentage of expression of antiapoptotic protein Bcl-2 (19.00% ± 2.40%) compared to the CTL (100.00% ± 2.80%, *p* < 0.001). However, both co- and posttreatment with KMUP-1 increased the percentage of Bcl-2 protein level, 152.00% ± 3.30% (*p* < 0.001) and 75.00% ± 2.50% (*p* < 0.05), respectively, compared to HFD mice.

Afterward, we examined whether KMUP-1 treatment affects the expression level of Atg7. As shown in Figure 4c, the HFD fed suppresses the Atg7 expression level compared to CTL (*p* < 0.05). Our findings indicated that HFD feeding significantly reduced the expression percentage of Atg7 protein compared to CTL, to 100.00 ± 3.50 and 52.25% ± 12.20%, respectively (*p* < 0.05). As expected, the expression level of Atg7 was elevated both in cotreatment KMUP-1 (153.98% ± 20.07%, *p* < 0.001) and in the posttreatment group (127.03% ± 13.74%, *p* < 0.05). Consistently, the expression level of downstream LC3-II is decreased in the HFD group (100.00% ± 1.95%, *p* < 0.05), compared with that of the CTL (64.10% ± 2.10%, *p* < 0.05) (see Figure 4d). Nevertheless, both the KMUP-1 cotreatment (114.30% ± 3.40%, *p* < 0.05) and the posttreatment groups (103.50% ± 2.60%, *p* < 0.05) resulted in a rise in the expression level of LC3-II. The LC3-II alteration could be associated with autophagosome formation at the initial stage of autophagy or autophagosome clearance at the late stage [10, 11]. These data suggest that KMUP-1 treatment exerts antiapoptosis effects by restoring impaired autophagy in the aortic tissue.

### 3.5. Dysregulated Serum Lipid Metabolism in HFD-Fed Mice Reversed by KMUP-1 Treatment

**Figure 5:**
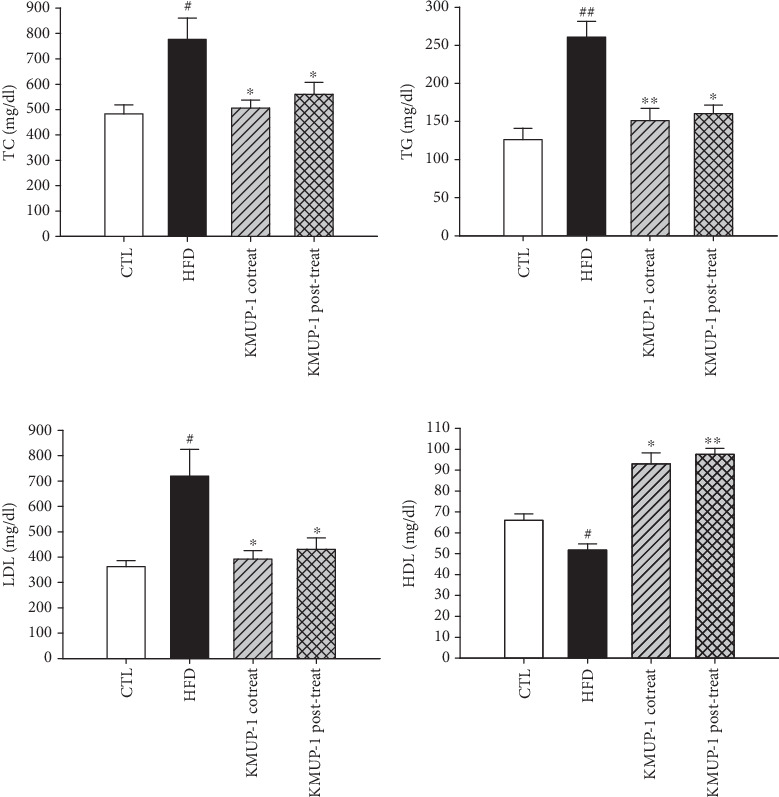
Serum lipid profile in the HFD-induced ApoE-KO mice after 12 weeks of KMUP-1 treatment. Serum levels of (a) total cholesterol (TC), (b) triglyceride (TG), (c) low-density lipoprotein cholesterol (LDL), and (d) high-density lipoprotein cholesterol (HDL) were measured with an automatic biochemical analyzer. Values were represented as mean ± SEM, *n* = 8. ⁣^#^*p* < 0.05 and ⁣^##^*p* < 0.001 versus CTL group. ⁣^∗^*p* < 0.01 and ⁣^∗∗^*p* < 0.01 versus HFD group.

In comparison with the CTL group, HFD fed significantly increased the TC content in the serum of ApoE-KO mice (Figure 5a; *p* < 0.05), and this was due to an increased formation of TG (Figure 5b; *p* < 0.01) and LDL (Figure 5c; *p* < 0.05) particles, as determined by an automatic biochemical analyzer. In contrast, the HDL particles were decreased in HFD-fed mice compared to CTL (*p* < 0.05). Administration with KMUP-1 co- and posttreatment significantly reduced TC, TG, and LDL levels (Figures 5a, 5b, and 5c). In addition, KMUP-1 co- and posttreatment also remarkably caused an increase in HDL particles (Figure 5d). In summary, these findings suggest that KMUP-1 alleviates HFD-enhanced atherosclerosis lesions and cardiac remodeling by restoring autophagic capacity.

## 4. Discussion

In this study, we investigated the impact of KMUP-1 on atherosclerotic lesions, inflammation, autophagy, and cardiac remodeling, as well as lipid metabolism indexes in an animal model of atherosclerosis, ApoE-KO mice, fed with an HFD. Our experiments showed that either a 12-week KMUP-1 cotreatment or a 4-week KMUP-1 posttreatment attenuated atherosclerosis lesions and cardiac remodeling in HFD-fed ApoE-KO mice. Here, KMUP-1 showed the ability to alleviate HFD-enhanced cardiovascular diseases through anti-inflammation and antiapoptotic effects, restoration of impaired autophagy in the aorta, and normalization of lipid metabolism in ApoE-KO mice. Furthermore, our findings indicated that KMUP-1 suppresses HFD-induced obesity and improves cardiac hypertrophy and dysfunction through its treatment.

Xanthine derivative KMUP-1, a phosphodiesterase inhibitor, was shown to improve hepatic lipid metabolism, reduce macrophage infiltration, and ameliorate steatohepatitis in an HFD-fed C57BL/6 mice model [21, 36]. Furthermore, we established an HFD-fed ApoE-KO mouse model that developed atherosclerosis combined with hypercholesterolemia. This research reveals that KMUP-1 co- and posttreatment could inhibit atherosclerotic plaque formation and atherosclerosis lesions by suppressing inflammation in HFD-fed mice (i.e., elevation of IL-1*β* and TNF-*α*). In addition, KMUP-1 treatment also reduced obesity and cardiac hypertrophy, as well as improved heart ventricular function (Figures 1 and 2). In our study, by using the ApoE-KO mouse model, we showed that KMUP-1 could potentially be used as a therapeutic intervention in the prevention of cardiac dysfunction associated with rapidly developing atherosclerosis. Since the ApoE-KO model shows a progressive decline in cardiac function, KMUP treatment, in a relatively short intervention window (12 weeks), reverses this trend. The powerful indicator, a statistically significant increase in FS and EF back toward the levels of a healthy CTL group, demonstrates that KMUP-1 is not just preventing further damage but is actively improving heart function. The physiological changes in FS and EF in the ApoE-KO mouse model are analogous to the cardiac functional decline seen in human patients with advanced atherosclerosis and heart failure [37]. Recently, increased aortic endothelial nitric oxide synthase (eNOS) and Akt protein expressions attenuated endothelial dysfunction in HFD-fed obese rats by time-restricted feeding [38]. Also, KMUP-1 is an eNOS enhancer, leading to lower hepatic fat and obesity enhanced by HFD feeding [21].

KMUP-1 has demonstrated anti-inflammatory characteristics, which are important for blocking inflammatory mediators and pathways in association with several inflammation-related diseases, including periodontitis [23], osteoarthritis [24], neuropathic pain [39], and allergic pulmonary vascular inflammation [40]. Thus, cardiovascular-related pathological effects and the risk of complications can be blocked by reducing inflammation. Further, KMUP-1 has been attributed to its effects on reducing oxidative stress by upregulation of heme oxygenase-1 and its ability to attenuate cardiac hypertrophy in a rat model [41, 42].

The most important observation of the present study was that KMUP-1 attenuated atherosclerosis development in ApoE-KO mice fed an HFD by restoring impaired autophagy. It is attractive to speculate that atherosclerosis induction is mediated by inflammation, cell apoptosis stimulation, and subsequent impairment of autophagic flux in atherosclerotic mice. Gu et al. demonstrated that oxidized LDL-treated THP-1 macrophages exhibited impaired autophagy flux, which was restored by the autophagy inducers rapamycin and curcumin nicotinate [43]. Our results reveal that HFD-fed mice have impaired vascular autophagy with augmented apoptotic protein expression. Notably, KMUP-1 co- and posttreatment rescued the impaired autophagy flux by significantly increasing the Atg7 level; this effect offers the antiapoptotic function to reverse atherosclerosis (Figures 3 and 4). Consistently, a fair number of studies report that disruptions in autophagy homeostasis are related to metabolic disorders, such as obesity, insulin resistance, diabetes mellitus, and atherosclerosis, no matter in both obese patients and animal models [44]. Excess adipose tissue is linked to an abnormal lipid profile and plays an essential role in all phases of the atherosclerotic process, a condition characterized by chronic lipid dysregulation and inflammation [45]. Here, we reveal that KMUP-1 co- and posttreatment rescue dysregulated serum lipid metabolism caused by HFD feeding (Figure 5). One of the supporting studies is that the inhibition of autophagy using small interfering RNA targeted to Atg7 increases proinflammatory gene expression (i.e., IL-1*β*, IL-6, and IL-8) in adipose tissue, thus suggesting that autophagy acts to suppress inflammation that is excessive during obesity [46]. Also, regarding lipid metabolism, KMUP-1 enhances adipogenesis by activating Protein Kinase A and G signaling, facilitating hormone-sensitive lipase activation, and promoting TG hydrolysis in 3T3-L1 adipocytes [47]. However, autophagy plays a dual role in cardiovascular diseases, acting as a double-edged sword. Basal and adaptive autophagy may slow the development of atherosclerotic plaques by reducing oxidative stress, inflammation, and lipid deposition. Conversely, excessive autophagy can lead to instability of plaques and cell death [8, 48]. Nevertheless, our previous studies reveal that optimizing autophagy modulation by another xanthine derivative treatment can be effective against cardiotoxicity and aortic aneurysm formation [33, 34]. Thus, maintaining autophagic homeostasis in cells through pharmacological intervention might be a therapeutic target for cardiovascular disease management. Limitations exist; although Atg7 and LC3-II are essential markers of autophagosome formation, they do not provide direct proof about the degradation of autophagic material, the full process of autophagosome formation, fusion with lysosomes, and degradation of their contents. To provide direct evidence of autophagic flux, it is necessary to establish additional experiments in our future work, including measurement of p62 accumulation and using lysosomal inhibitors as a gold standard method for evaluating autophagic flux.

## 5. Conclusion

**Figure 6:**
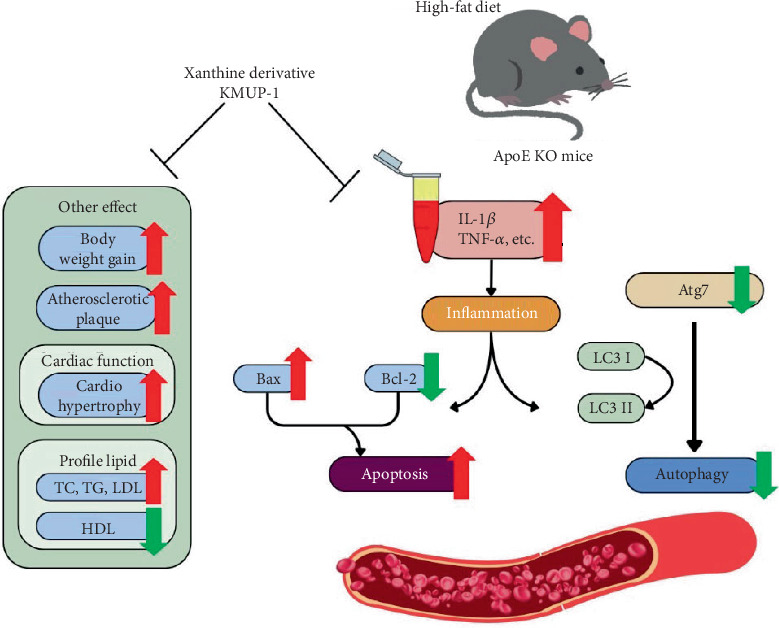
Schematic illustration of KMUP-1 alleviates atherosclerosis and cardiac remodeling by restoring impaired autophagy.

Our self-developed xanthine derivative KMUP-1 provides promise in the inhibition of atherosclerosis and cardiac remodeling through improving impaired autophagy, including reducing inflammation and suppressing apoptosis (Figure 6). Our findings deliver potential candidates for therapeutic interventions targeting cardiovascular diseases associated with atherosclerotic plaque formation and arterial wall thickening. Further studies are required to thoroughly assess its target gene and safety profile in VSMC-specific Atg7-KO mice [12] and miniature swine models [49] before clinical trials.

## Figures and Tables

**Table 1:** Cardiac function indicators in ApoE-KO mice fed with HFD.

**Parameter**	**CTL**	**HFD**	**KMUP-1 + cotreat**	**KMUP-1 + posttreat**	**p** ** value (co- and posttreat vs. HFD)**
LVEDD (mm)	3.51 ± 0.20	4.10 ± 0.25^#^	3.55 ± 0.29^∗^	3.62 ± 0.30^∗^	< 0.05
LVESD (mm)	2.35 ± 0.09	2.92 ± 0.18^#^	2.41 ± 0.11^∗^	2.50 ± 0.17^∗^	< 0.05
FS (%)	39.74 ± 1.33	30.08 ± 1.78^##^	38.45 ± 2.51^∗^	36.23 ± 2.67^∗^	< 0.05
EF (%)	76.36 ± 0.94	63.88 ± 1.77^#^	73.77 ± 1.83^∗^	70.58 ± 3.18^∗^	< 0.05

*Note:* Values were as mean ± SEM. Each group has *n* = 8.

Abbreviations: EF, ejection fraction; FS, fractional shortening; LVEDD, left ventricular end-diastolic dimension; LVESD, left ventricular end-systolic dimension.

^#^ *p* < 0.05 and ⁣^##^*p* < 0.01 versus CTL group. ⁣^∗^*p* < 0.05 versus HFD group.

## Data Availability

The data that support the findings of this study are available from the corresponding author (J-L.Y.) upon reasonable request.

## Supporting Information

Supporting Information 1 Figure S1: KMUP-1 alleviates body weight gain in the high-fat diet-induced ApoE-KO mice. (A) Representative photographs of mice were fed for 12 weeks with a normal diet (CTL), high-fat diet (HFD), cotreatment KMUP-1 (KMUP-1 cotreat) combined with HFD, and posttreatment KMUP-1 (KMUP-1 posttreat) combined with HFD. (B) Comparison of the mean body weights of each group in a time-dependent manner. Values were represented as *mean* ± *SEM*, *n* = 8. ⁣^###^*p* < 0.001 versus CTL group. ⁣^∗∗^*p* < 0.01 versus HFD group.

Supporting Information 2 Figure S2: KMUP-1 ameliorates cardiac hypertrophy in ApoE-KO mice fed with HFD. (A) Representative images of the heart sections were performed using hematoxylin and eosin staining. (B, C) Comparison of the (B) whole heart weight to body weight and the (C) whole heart weight to tibia length of mice. All scale bars show 1 mm. Values were represented as *mean* ± *SEM*, *n* = 8. ⁣^###^*p* < 0.001 versus CTL group. ⁣^∗^*p* < 0.05 and ⁣^∗∗∗^*p* < 0.001 versus HFD group.

Supporting Information 3 Figure S3: KMUP-1 improves left ventricular function in ApoE-KO mice fed with HFD. Measurement has been taken by echocardiography. (A) M-mode echocardiogram images were represented from individual groups. The values of (B) LVEDD (left ventricular end-diastolic dimension) and (C) LVESD (left ventricular end-systolic dimension) and (D, E) the percentage of FS (fractional shortening) and EF (ejection fraction) were calculated by echocardiographic parameters. The data represent the average value of all test animals in each group. Values were as *mean* ± *SEM*, *n* = 8. ⁣^#^*p* < 0.05 and ⁣^##^*p* < 0.01 versus CTL group. ⁣^∗^*p* < 0.05 versus HFD group.
