# Supplementary figures and images for: Antiatherogenic and Cardioprotective Effects of a Xanthine Derivative KMUP-1 in ApoE Knockout Mice
Source: Cardiovasc Ther. 2025 Oct 28;2025:8419343. doi: 10.1155/cdr/8419343 (PMC12585789; doi:10.1155/cdr/8419343)

**A**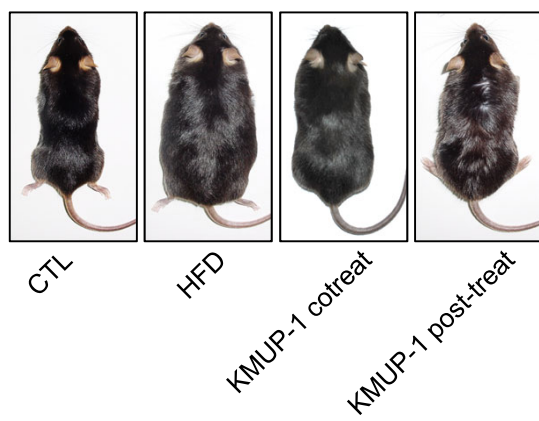**B**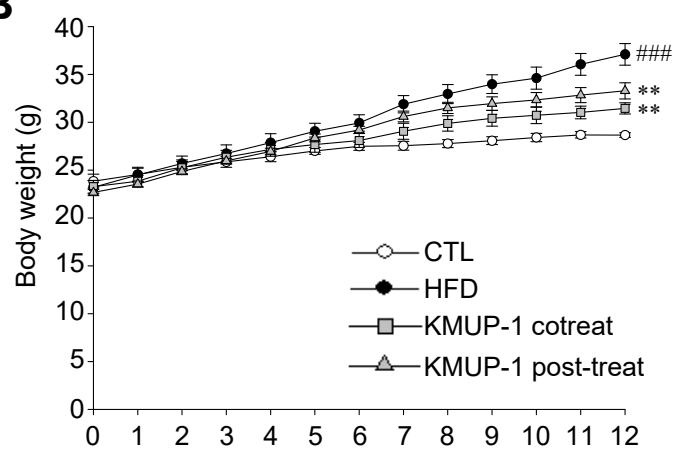

Supplement: Supporting Information 1 — Figure S1: KMUP-1 alleviates body weight gain in the high-fat diet-induced ApoE-KO mice. (A) Representative photographs of mice were fed for 12 weeks with a normal diet (CTL), high-fat diet (HFD), cotreatment KMUP-1 (KMUP-1 cotreat) combined with HFD, and posttreatment KMUP-1 (KMUP-1 posttreat) combined with HFD. (B) Comparison of the mean body weights of each group in a time-dependent manner. Values were represented as mean ± SEM, n = 8. ⁣###p < 0.001 versus CTL group. ⁣∗∗p < 0.01 versus HFD group. [file 8419343.f1.pdf]

**A**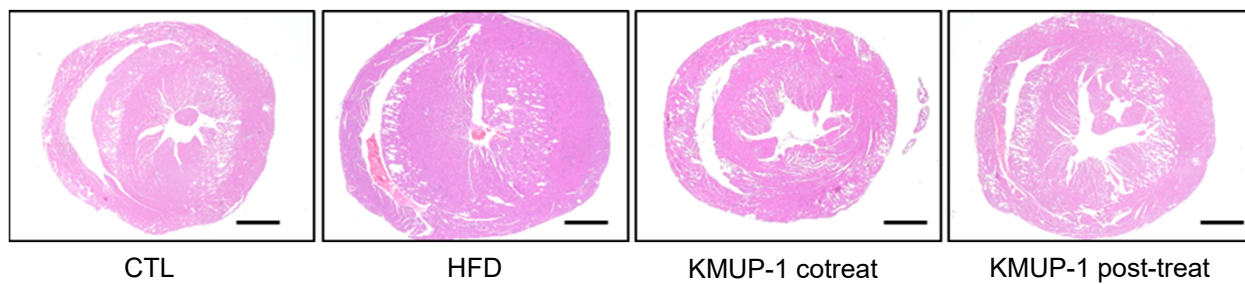**B**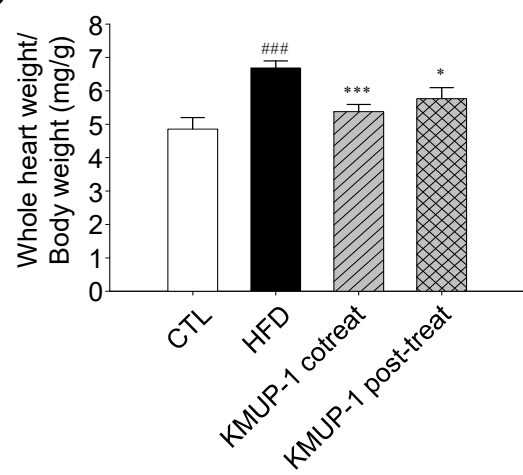**C**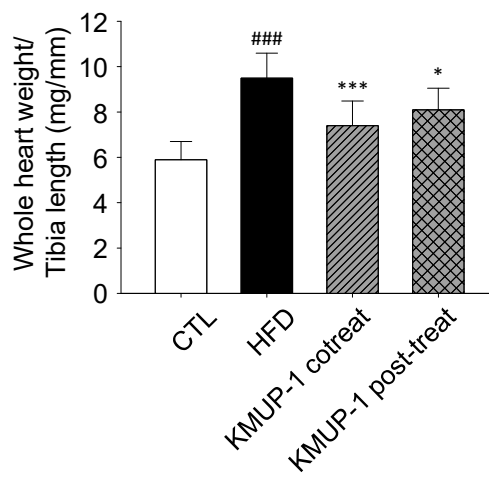

Supplement: Supporting Information 2 — Figure S2: KMUP-1 ameliorates cardiac hypertrophy in ApoE-KO mice fed with HFD. (A) Representative images of the heart sections were performed using hematoxylin and eosin staining. (B, C) Comparison of the (B) whole heart weight to body weight and the (C) whole heart weight to tibia length of mice. All scale bars show 1 mm. Values were represented as mean ± SEM, n = 8. ⁣###p < 0.001 versus CTL group. ⁣∗p < 0.05 and ⁣∗∗∗p < 0.001 versus HFD group. [file 8419343.f2.pdf]

**A**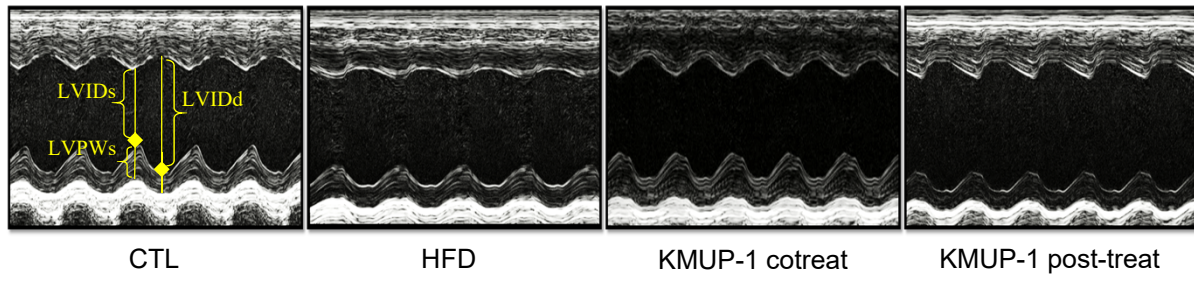**B**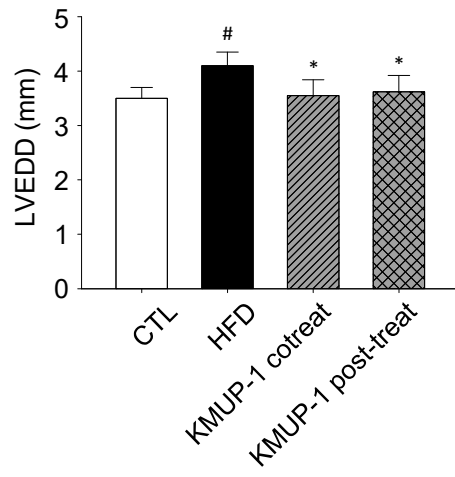**C**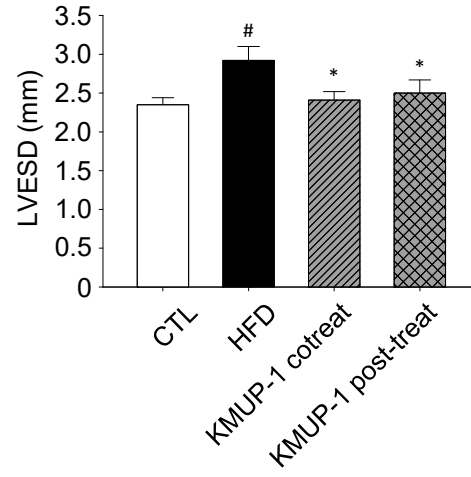**D**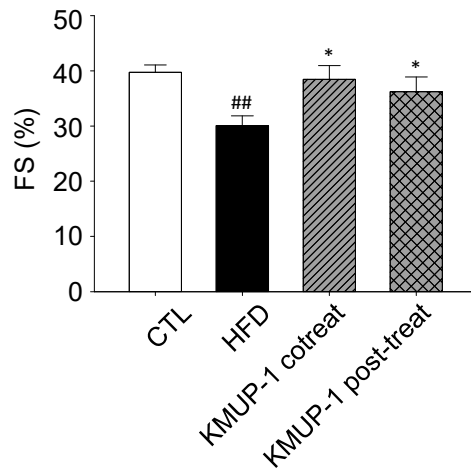**E**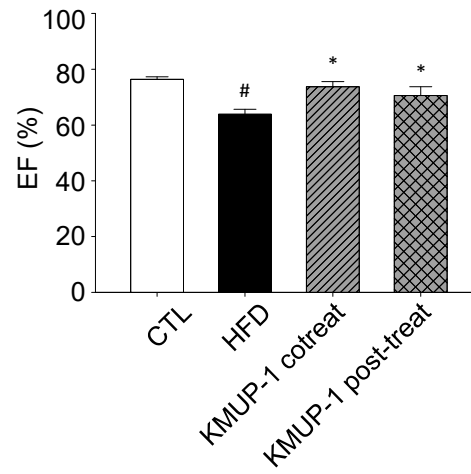

Supplement: Supporting Information 3 — Figure S3: KMUP-1 improves left ventricular function in ApoE-KO mice fed with HFD. Measurement has been taken by echocardiography. (A) M-mode echocardiogram images were represented from individual groups. The values of (B) LVEDD (left ventricular end-diastolic dimension) and (C) LVESD (left ventricular end-systolic dimension) and (D, E) the percentage of FS (fractional shortening) and EF (ejection fraction) were calculated by echocardiographic parameters. The data represent the average value of all test animals in each group. Values were as mean ± SEM, n = 8. ⁣#p < 0.05 and ⁣##p < 0.01 versus CTL group. ⁣∗p < 0.05 versus HFD group. [file 8419343.f3.pdf]
